# Biomarkers and neurobiology of schizophrenia

**DOI:** 10.1007/s00406-012-0340-9

**Published:** 2012-06-29

**Authors:** Peter Falkai, Hans-Jürgen Möller

**Affiliations:** Department of Psychiatry and Psychotherapy, Ludwigs-Maximilians-University Munich, Nußbaumstr. 7, 80336 Munich, Germany

Dedicated to the neurobiology of schizophrenia, this issue opens with an immunohistochemical study on changes in hypothalamic VGF expression by Busse et al. [1]. The authors investigated the relation of increased levels of the VGF fragment 23–62 in CSF to changes in hypothalamic VGF expression. They found first evidence for diminished hypothalamic VGF levels in schizophrenia, particularly significant in subjects without metabolic syndrome. Thus, VGF, apart from effects on synaptic plasticity and neurogenesis, might be linked to schizophrenia-related alterations in energy homeostasis. In the search for further possible schizophrenia biomarkers, Vasic et al. [2] evaluated different online databases with regard to the clinical relevance of CSF candidate markers as potential surrogates for disease activity, prognosis assessment, and predictors of treatment response. Analyses of metabolites in CSF showed altered glutamatergic neurotransmission, monoamine, and cannabinoid metabolism, added by hints on dysregulated neuroprotective and neurodevelopmental processes. In conclusion, the authors claim a better characterization of psychopathological profiles with respect to different schizophrenia phases in longitudinal investigations plus implementing different approaches of proteomics and rigorously adhering to standard procedures according to international CSF guidelines to improve the quality of CSF studies in schizophrenia.

Again focussing on the role of protein modifications such as phosphorylation, the work group of Bahn [3] intends increasing insights into schizophrenia etiology and the mechanism of action of antipsychotic medications. Concordantly, the group conducted a very interesting first large-scale mass spectrometry-based analysis of phosphoproteins in serum from 22 anti-psychotic-naïve first-onset paranoid schizophrenia patients compared to 33 controls. They were able to identify 710 phosphopeptides corresponding to 164 non-redundant proteins. They hope that in the long term differentially phosphorylated proteins in diseased or treated tissues might serve as biomarkers which could be implemented into a blood-based multiplex immunoassay test to diagnose schizophrenia. Since the involvement of the immune system has been shown to be relevant for the schizophrenia pathophysiology by several studies, Krause et al. [4] measured the demonstrably affected monocyte-derived cytokines TNF-α, IL-6, and IL-10 in schizophrenia inpatients. While in an unstimulated condition intracellular monocytic IL-6 levels at baseline were significantly lower in schizophrenic patients than controls, after mimicking an infection via stimulation with lipopolysaccharide or poly I/C, monocytic intracellular IL-6 production tended to increase in the patient group. These results seem to strengthen the hypothesis of a dysfunction of the monocytic system and impairment of especially the pro-inflammatory immune response in schizophrenia.

Application of *N*-methyl-D-aspartate receptor (NMDAR) antagonists is a well-established pharmacological model of psychotic symptoms. Nagels et al. [5] further investigated symptoms in 15 healthy subjects receiving a continuous subanesthetic *S*-ketamine infusion while their cortical activation was measured with fMRI. During scanning, the subjects underwent an overt word generation task and psychopathological symptoms were assessed with the Positive and Negative Syndrome Scale (PANSS). Ketamine effects on psychopathology resulted in difficulties in abstract thinking, lack of spontaneity, and conversation flow plus formal thought disorder, while verbal fluency performance remained unaffected. All of these findings were positively correlated with fMRI activation measures. These results confirm the hypothesis of an NMDAR dysfunction in the pathophysiology of schizophrenia. Despite an evidential link between reduced glutamate-based plasticity and dysconnectivity in schizophrenia patients, information on the impact of glutamate-dependent long-term depression (LTD)-like cortical plasticity on inter-hemispheric connectivity is sparse. Concordantly, Hasan et al. [6] investigated LTD-like cortical plasticity following excitability-diminishing cathodal transcranial direct current stimulation (tDCS) of the left primary motor cortex (MI) and its effect on the non-stimulated right MI in schizophrenia patients compared to healthy controls. On the stimulated hemisphere, both groups showed an increase of resting motor thresholds (RMT) while decreased motor-evoked potential (MEP) sizes more often occurred in healthy controls. On the non-stimulated hemisphere, only the control group showed increased RMTs and decreased MEPs. The results point to an association between LTD-like cortical plasticity and inter-hemispheric connectivity and highlight the impact of plasticity on connectivity in schizophrenia patients.

For being an important treatment for catatonia, Raveendranathan et al. [7] retrospectively analyzed the number of administered electroconvulsive therapy (ECT), seizure threshold, failure in achieving adequate seizures plus clinical signs for the disorder in all respectively diagnosed patients admitted to their department within 12 months. Response to 4 ECT sessions was considered as fast, to 5 or more sessions as slow. The authors found a significantly lower duration of catatonia plus higher catatonia score at presentation in fast responders, in whom presence of waxy flexibility predicted faster response, whereas presence of echophenomena predicted slow response. As another aspect of treatment, Rabovsky et al. [8] randomly assigned inpatients to a bifocal diagnosis-mixed group using psychoeducation or a non-specific intervention. Effects were measured at baseline plus 3- and 12-month follow-ups. As regards readmission, compliance, and clinical variables, after 3 months the psychoeducationally treated group showed significantly better compliance and lower suicide rate than controls, whereas most other outcome variables showed merely insignificant advantages. Anyhow, since this measure is easily implementable, the authors recommend enhanced clinical application in clinical practice.

In the final article, Helmchen [9] addresses ethical, legal, and professional questions concerning research with mentally ill persons, particularly the informed consent process, the relationship between benefits and risks plus standards and criteria of benefits and risks and/or burdens of research interventions. There is a demand of protecting participants of clinical research and preserving trust of both patients and public via strict adherence to the ethical rules along his listed set of recommendations.
